# Rat lungworm survives winter: experimental overwintering of *Angiostrongylus cantonensis* larvae in European slugs

**DOI:** 10.1017/S0031182023000781

**Published:** 2023-09

**Authors:** Lucia Anettová, Anna Šipková, Elena Izquierdo-Rodriguez, Vivienne Velič, David Modrý

**Affiliations:** 1 Department of Botany and Zoology, Faculty of Science, Masaryk University, Brno, Czech Republic; 2 Instituto Universitario de Enfermedades Tropicales y Salud Pública de Canarias, Universidad de La Laguna, La Laguna, Canary Islands, Spain; 3 Department of Obstetrics and Gynecology, Pediatrics, Preventive Medicine and Public Health, Toxicology, Legal and Forensic Medicine and Parasitology, Universidad de La Laguna, La Laguna, Canary Islands, Spain; 4 University of Veterinary Sciences Brno, Palackého tř. 1946/1, 612 42 Brno, Czech Republic; 5 Institute of Parasitology, Biology Center of Czech Academy of Sciences, Ceske Budejovice, Czech Republic; 6 Department of Veterinary Sciences, Faculty of Agrobiology, Food and Natural Resources/CINeZ, Czech University of Life Sciences Prague, Prague, Czech Republic

**Keywords:** *Angiostrongylus cantonensis*, invasive nematode, *Limax maximus*, overwintering

## Abstract

The rat lungworm *Angiostrongylus cantonensis* is a metastrongyloid nematode that causes neurological disorders in its accidental hosts, including humans. This invasive pathogen is native to Southeast Asia and adjacent regions and is gradually expanding its distribution to tropical and subtropical areas with new foci discovered near temperate regions. The parasite has a complex life cycle with a range of gastropods serving as intermediate hosts. A broad spectrum of poikilotherm vertebrates and invertebrates can serve as paratenic hosts. Since it has already been demonstrated that other, non-zoonotic metastrongyloids can survive in their intermediate hosts during the winter, the aim of our study was to evaluate the survival of *A. cantonensis* third-stage larvae in experimentally infected slugs (*Limax maximus*) kept at 4.5–7°C for 60 days. Third-stage larvae of *A. cantonensis* survived the period of low temperature and remained capable of infecting definitive hosts (laboratory rats) afterwards, even though their numbers dropped significantly. These results suggest that further spread to higher latitudes or altitudes is possible in areas with sufficient abundance of definitive hosts, since low winter temperatures are not necessarily an obstacle to the spread of the parasite.

## Graphical abstract

**Figure:**
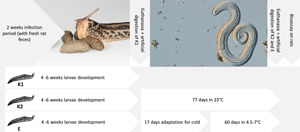

## Introduction

The rat lungworm, *Angiostrongylus cantonensis* (Chen, 1935) is a metastrongyloid nematode widely known for its zoonotic potential and unusually broad range of paratenic and accidental hosts. Besides humans and domestic animals as its well-described accidental hosts, the parasite has been reported as causing severe neurological disorders in wildlife. In all these accidental hosts, the parasite can lead to paralysis or death (Kuberski and Wallace, 1979; Lindo *et al*., 2004; Monks *et al*., 2005; Burns *et al*., 2014, Cowie, 2017; Odani *et al*., 2021). In most cases, humans get infected with *A. cantonensis* by eating raw or undercooked intermediate or paratenic hosts that contain infectious L3 larvae (Wang *et al*., 2012). *Angiostrongylus cantonensis* originated in Southeast Asia and adjacent regions (Chen, 1935; Lv *et al*., 2018), but is now well established in tropical and subtropical regions around the globe (Prociv and Carlisle, 2001; Caldeira *et al*., 2007; Stockdale Walden *et al*., 2017). In temperate regions, 6 species of the genus *Angiostrongylus* and 4 other species of closely related genera (*Rodentocaulus* spp., *Stefanskostrongylus* spp.) have been described (Cowie, 2019), and only one of them (*A. dujardini*) is known to harm animals other than its definitive hosts (Graille *et al*., 2015; Eleni *et al*., 2016). *Angiostrongylus cantonensis* cycles between definitive (several rat species mainly of the tribe Rattini) and intermediate (terrestrial and aquatic gastropods) hosts (Alicata, 1965, Yong and Eamsobhana, 2013). In addition, various poikilotherm vertebrates as well as invertebrates serve as paratenic hosts (Wallace and Rosen, 1967; Ash, 1968; Radomyos *et al*., 1994; Wang *et al*., 2018; Chaisiri *et al*., 2019, Turck *et al*., 2022).

Factors contributing to the emergence of *A. cantonensis* and its expanding distribution include climate change, global transport and the resulting facilitated movement of hosts (rodents and gastropods) and, finally, changes in population dynamics of hosts and cultural habits in endemic areas (York *et al.*, 2015; Cowie, 2017). Findings of new *A. cantonensis* foci in the last 2 decades show not only continuous extension of its distribution, but also an expansion into temperate regions of Europe and USA (Campbell and Little, 1988; Foronda *et al*., 2010; Paredes-Esquivel *et al*., 2019; Walden *et al*., 2021; Delgado-Serra *et al*., 2022; Galán-Puchades *et al*., 2022). York *et al*. (2014) predicted an increase of suitable habitats for *A. cantonensis* within Europe.

There is an extensive range of metastrongyloid nematode species that thrive in the temperate realm. (Gonzáles *et al*., 2007; Ferdushy *et al*., 2009; Majoros *et al*., 2010; Traversa and Di Cesare, 2014; Čabanová *et al*., 2018; Morgan *et al*., 2021). The ability of their larvae to survive or even continue development in gastropods during the winter months in temperate climate has been experimentally demonstrated in *Troglostrongylus brevior* (Gerichter, 1948; Morelli *et al*., 2020) and *Aelurostrongylus abstrusus* (Morelli *et al*., 2021). To our knowledge, there have been no studies of *A. cantonensis* survival in overwintering intermediate hosts. We hypothesized and experimentally tested that *A. cantonensis* larvae can survive low winter temperatures in gastropods, which may be one of the key factors enabling their future expansion deeper into temperate regions.

## Materials and methods

The experimental strain of *A. cantonensis* was obtained in 2017 in Fatu Hiva, French Polynesia and is maintained in laboratory conditions circulating between Wistar rats and experimental gastropods (*Subulina octona* and *Biomphalaria glabrata*). The identity of the isolate was confirmed by the morphology of adult nematodes obtained from infected rats as well as by cox1 sequencing, with the haplotype identified as part of *A. cantonensis* clade 2 (Červená *et al*., 2019).

**Figure 1.**
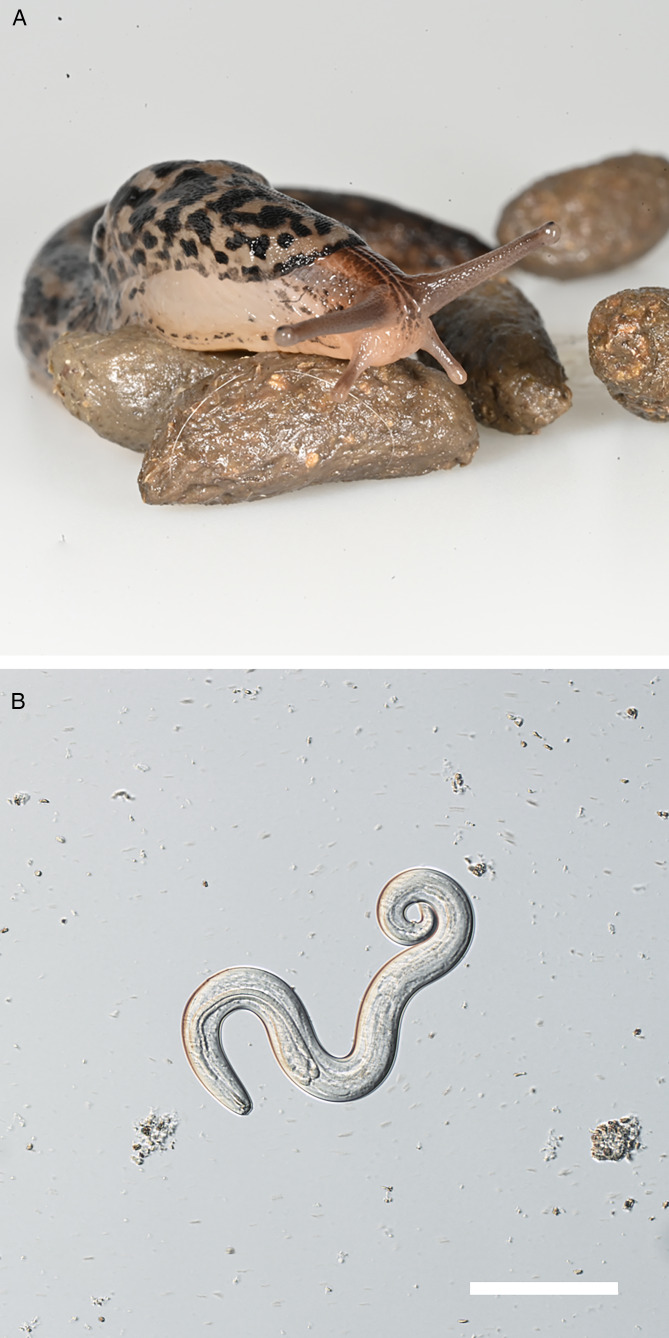
Hosts and developmental stages of *A. cantonensis* in described experiments. **A.** Slugs *Limax maximus* feeding on fresh rat feces; **B.** *Angiostrongylus cantonensis* L3 larva collected from artificially digested slugs, scale bar = 100 *μ*m.

Adult great grey slugs (*Limax maximus* – LM) were collected from a private garden near Brno, Czech Republic during Autumn 2021. Slugs were kept in plastic boxes containing 8–10 individuals at 20–24°C throughout the experiment except for adaptation to cool temperature and the overwintering period of the experimental group. Boxes containing slugs were equipped with shelters and moss, and coconut soil was used as a bedding. Slugs were checked daily and fed with fresh lettuce and fish flakes every other day. One experimental group (E, 34 individuals) and 2 control groups (K1, 22 individuals and K2; 30 individuals) with a total of 86 LM individuals were infected by offering approximately 1 gram of fresh rat feces (maximum 12 hours old) to slugs in all experimental and control groups for 2 weeks (Fig. 1A). The number of larvae per gram of feces was determined by modified Baermann larvoscopy, such that approximately 40 000 L1 larvae per experimental box were provided during the 2-week period.

Experimental group E contained 34 slugs. After the exposure to rat feces containing L1 larvae, all slugs were left for 4 weeks to allow larvae to develop to the third stage. After that period, slugs in group E were gradually adapted to the decreasing temperature by exposing them to 15°C for two 5-day periods with a 7-day break between them at room temperature. After this adaptation period, slugs from group E were deprived of food for 3 days to empty the digestive tract and placed in a laboratory cool room with temperature between 4.5 and 7°C for 60 days. These conditions simulated the natural conditions in which these slugs overwinter, hidden in a shelter or deep underground, where the temperature rarely dip below 0°C (South, 2012). During this period, the slugs were kept undisturbed and without food, except for weekly checks. All the slugs from group E exhibited typical signs of hibernation, i.e. clustered together with no apparent physical activity. After this period, all the slugs from groups E and K2 were weighed, sacrificed by decapitation and individually digested artificially (i.e. 0.3 g pepsin and 100 mL 0.7% HCl on a magnetic stirrer set to 200 RPM and 37°C) (Modrý *et al*., 2021). Motile L3 larvae were isolated, counted and used for the confirmatory bioassay on 2 infection-naive Wistar laboratory rats to assess their infectivity. Each rat was infected with 60 live motile L3 larvae *via* orogastric tube under general inhalation anaesthesia with diethyl ether. After 45 days post infection, Baermann larvoscopy of rat feces was performed to detect L1 larvae.

Slugs from group K1 (22) were used for confirmation of infection success. These slugs were sacrificed 4 weeks after the infection period, artificially digested as described and the number of larvae per slug was counted. Throughout the experiments, larvae were microscopically confirmed as *A. cantonensis* based on typical morphology, i.e. tail length, shape of the termination of the tail and oesophagus length (Ash, 1970); possible presence of other metastrongylids was excluded using the same criteria (Diakou *et al*., 2016).

Slugs from group K2 (30) did not overwinter and remained at room temperature (20–24°C) throughout the experimental period, to check that *A. cantonensis* larvae survive in this slug species for this amount of time at room temperature. Slugs were checked daily and fed every other day as before the experiment. The remains of slugs from this group that died spontaneously less than 4 weeks before the end of the experiment were immediately examined for *A. cantonensis* larvae by artificial digestion. The slugs from K2 that died spontaneously more than 30 days before the end of experiment were excluded. Remaining slugs were sacrificed 4 weeks after the infection period, artificially digested as described above and the number of larvae per individual slug was counted.

Statistical analysis was done using ANOVA and the Tukey HSD test. The average, *P* value and standard deviation were calculated in R Studio and Microsoft Excel was used to generate a boxplot graph.

## Results

All 34 slugs from the experimental (i.e. overwintering) group E survived the entire experiment. In the control group K1 (22 individuals), there were no mortalities either since the slugs were sacrificed just after the larval development to L3. Of 30 slugs in control group K2, 21 survived the entire experiment, 9 of them died during the last month of the experiment and were excluded from the experiment. Six more slugs from K2 group died spontaneously in the last month of the experiment and were processed as those that were sacrificed. The average weight of slugs at the end of the experiment in groups K1, K2 and E was 2.0, 2.2 and 2.3 g, respectively.

**Figure 2.**
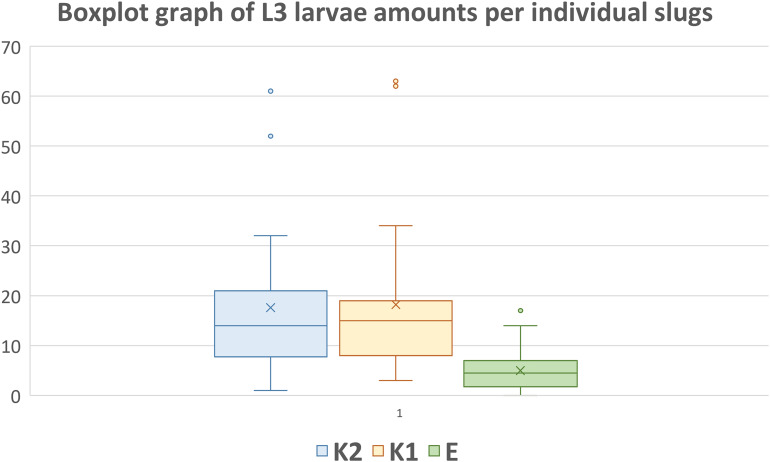
Boxplot graph of numbers of third-stage larvae collected from each slug. The X marks the average value. The median is indicated by a line across the box. The whiskers on box plots show the ranges of Q1 and Q4 up to the most extreme data points. K1 and K2 are control groups, E is the experimental overwintering group of slugs.

In the overwintering group (E), 0–17 motile third-stage metastrongyloid larvae (Fig. 1B) were collected from individual slugs (an average of 4.97 larvae per slug). In control groups K1 and K2, average numbers of larvae retrieved were 17.6 and 18.4, respectively, with a maximum of 61 larvae per slug in K1 and 63 larvae per slug in K2. Detailed information on the number of larvae in each slug are given in Table 1. The difference of L3 survival between the controls and overwintering group is significant (F_2,74_ = 20.05, *P* < 0.001) according to the Tukey HSD test and the difference between the 2 control groups is negligible (*P* = 0.95). Figure 2 is a boxplot graph of numbers of the third-stage larvae collected in each group.

**Table 1.** Numbers of live motile L3 of *A. cantonensis* retrieved from each individual slug *Limax maximus* from control groups K1 and K2 and in overwintering group E. The first six slugs from K2 group died spontaneously during the last month of the experiment.

K1		K2		E	
Slug no.	Number of larvae	Slug no.	Number of larvae	Slug no.	Number of larvae
1	20	†1	20	1	5
2	15	†2	62	2	3
3	2	†3	15	3	14
4	12	†4	5	4	5
5	20	†5	15	5	17
6	25	†6	4	6	5
7	19	7	9	7	0
8	1	8	17	8	1
9	3	9	11	9	2
10	8	10	10	10	2
11	13	11	3	11	1
12	61	12	15	12	6
13	11	13	12	13	3
14	10	14	18	14	7
15	24	15	6	15	5
16	52	16	14	16	11
17	20	17	7	17	4
18	7	18	34	18	13
19	6	19	24	19	1
20	8	20	63	20	1
21	18	21	18	21	1
22	32			22	11
				23	9
		9 slugs from K2 died spontaneously before January 2022		24	2
				25	0
				26	7
				27	2
				28	2
				29	10
				30	1
				31	5
				32	6
				33	2
				34	5
AVERAGE	17.6	AVERAGE	18.19	AVERAGE	4.97

Both Wistar laboratory rats that served as positive controls for the infectivity of larvae retrieved from the overwintering group E had L1 in their feces on day 45 post infection.

## Discussion

Our study confirmed the ability of third-stage larvae of *A. cantonensis* to survive winter conditions in gastropods native to temperate Europe and remain infective for the definitive hosts. This result, together with the relatively long patent period in rats (Mackerras and Sandars, 1955), suggests that the range could further extend in Europe into areas with milder winters and sufficient abundance of definitive hosts.

Even though the collected larvae were infective for rats as definitive hosts, we observed a decrease in numbers of live motile larvae in slugs during the overwintering period (average 4.97 larvae per slug in group E compared to 18.19 in group K2). We chose *L. maximus* as a model species since it is a synanthropic slug with a lifespan of 2–3 years, naturally overwintering in shelters where the temperature almost never falls below 0°C (South, 2012). The highest mortality observed in group K2, which was kept at room temperature, may be attributable to their maladaptation to captive conditions, since overwintering is natural for these slugs. LM has been described as an intermediate host suitable for other metastrongyloid nematodes (Conboy *et al*., 2017), although it usually carries relatively low numbers of L3 larvae (Ferdushy *et al*., 2009). Other slug species in temperate Europe reported to host metastrongyloids also showed low abundance of larvae, barely reaching 100 larvae per individual (Lange *et al*., 2018). For comparison, the numbers of *A. cantonensis* L3 recovered from tropical slugs may reach hundreds to thousands (or even millions) of larvae, depending on the species (Heyneman and Lim, 1967; Hollingsworth *et al*., 2013; Kim *et al*., 2014; Rollins *et al*., 2021). Environmental temperature has an impact on activity of L1 larvae and their ability to develop in intermediate hosts (Ishii, 1984; Lv *et al*., 2006). However, the massive load of *A. cantonensis* larvae in some tropical snails and slugs could also be related to differences in behaviour, food preferences and overall ecology of intermediate and definitive hosts.

Previous studies have shown that *A. cantonensis* larvae cannot develop to the third stage at less than 15°C (Ishii, 1984; Lv *et al*., 2006), which is very similar to the case of *A. vasorum* (Ferdushy *et al*., 2010). However, our results confirmed that already developed L3 larvae survive and maintain their infectivity like the latter species, which is widely distributed in carnivores in temperate Europe (Fuehrer *et al*., 2021; Tieri *et al*., 2021).

Our experiments indicate that once an infected mollusc as an intermediate host survives cold winter temperature, larvae of *A. cantonensis* most likely will stay infective within it for several months. This result, together with the relatively long patent period in rats (Mackerras and Sandars, 1955) suggests further possible range expansion to higher latitudes or altitudes in areas with sufficient abundance of the definitive hosts as the low winter temperatures are not necessarily an obstacle to the spread of the parasite (until reaching certain very high latitude or altitude points).

## Data Availability

All data generated and analysed during this study are included in this published article.
